# Characterization of Triptolide-Induced Hepatotoxicity by Imaging and Transcriptomics in a Novel Zebrafish Model

**DOI:** 10.1093/toxsci/kfx144

**Published:** 2017-07-20

**Authors:** Adriaan D. Bastiaan Vliegenthart, Chunmin Wei, Charlotte Buckley, Cécile Berends, Carmelita M. J. de Potter, Sarah Schneemann, Jorge Del Pozo, Carl Tucker, John J. Mullins, David J. Webb, James W. Dear

**Affiliations:** * Edinburgh University/BHF Centre for Cardiovascular Science, The Queen’s Medical Research Institute, Edinburgh EH16 4TJ, UK; † Center for Drug Evaluation, China Food and Drug Agency, Beijing 100083, China; ‡ Easter Bush Pathology, Royal (Dick) School of Veterinary Studies, The University of Edinburgh, Easter Bush Campus, Roslin, Midlothian EH25 9RG, UK;; § Biomedical Research Resources, The College of Medicine and Veterinary Medicine, The University of Edinburgh, Edinburgh EH16 4TJ, UK

**Keywords:** triptolide, hepatotoxicity, zebrafish, microRNA-122, imaging, nitric oxide synthase

## Abstract

Triptolide is a vine extract used in traditional Chinese medicines and associated with hepatotoxicity. *In vitro* data suggest that inhibition of RNA synthesis may be the mechanism of toxicity. For studying drug-induced liver injury the zebrafish has experimental, practical and financial advantages compared with rodents. The aim of this study was to explore the mechanism of triptolide toxicity using zebrafish as the model system. The effect of triptolide exposure on zebrafish larvae was determined with regard to mortality, histology, expression of liver specific microRNA-122 and liver volume. Fluorescent microscopy was used to track toxicity in the Tg(*-2.8lfabp*:GFP)^as3^ zebrafish line. Informed by microscopy, RNA-sequencing was used to explore the mechanism of toxicity. Triptolide exposure resulted in dose-dependent mortality, a reduction in the number of copies of microRNA-122 per larva, hepatocyte vacuolation, disarray and oncotic necrosis, and a reduction in liver volume. These findings were consistent across replicate experiments. Time-lapse imaging indicated the onset of injury was 6 h after the start of exposure, at which point, RNA-sequencing revealed that 88% of genes were down-regulated. Immune response associated genes were up-regulated in the triptolide-treated larvae including nitric oxide synthase. Inhibition of nitric oxide synthase increased mortality. Triptolide induces hepatotoxicity in zebrafish larvae. This represents a new model of drug-induced liver injury that complements rodents. RNA sequencing, guided by time-lapse microscopy, revealed early down-regulation of genes consistent with previous *invitro* studies, and facilitated the discovery of mechanistic inflammatory pathways.

Traditional Chinese Medicines (TCMs), including herbal drugs, have been used for thousands of years (Zhang *etal.*, 2012). Currently, TCMs are used around the world with 70%–95% of the population in certain developing countries relying on it for their primary medical care (Parveen *etal.*, 2015; Robinson and Zhang, 2011). In the United States, in 2004, around 1 in 5 adults reported taking a herbal product during the past 12 months (Barnes *etal.*, 2004). Despite this high global use of TCM, evidence for efficacy is limited (Bent, 2008; Parveen *etal.*, 2015). On the other hand, some TCMs clearly cause toxicity (Bunchorntavakul and Reddy, 2013; Teschke *etal.*, 2012). As there is little evidence of efficacy, it is important to understand safety/toxicity, in order to protect users from unacceptable harms.

An archetypal example of a medicinal plant with a long history of use in TCM is *Tripterygium wilfordii* Hook F, also known as “thunder duke vine” (Kupchan *etal.*, 1972). Its major active ingredient is triptolide (TP), a diterpene triepoxide. TP has been reported to have multiple pharmacological activities, including neuroprotective, anti-inflammatory and contraceptive effects (Chen, 2001; Liu, 2011; Zheng *etal.*, 2013; Ziaei and Halaby, 2016). Unfortunately, the relatively high incidence of toxicity, predominately to the liver, and a narrow therapeutic window, has limited the clinical development of TP (Li *etal.*, 2014). A synthetic pro-drug of TP, F60008, has entered phase 1 clinical trials in patients with advanced solid tumors. However, out of 20 study participants, 2 died. One subject died without clear cause and the other most likely died from neutropenic sepsis. Further development of F60008 was stopped due to these 2 deaths and marked variability in its pharmacokinetics (Kitzen *etal.*, 2009).

TP is extensively metabolized, with <1% of a single dose being recovered in bile, urine or feces within 48 h (Shao *etal.*, 2007). After administration of TP to rats, its concentration was at least 3-fold higher in liver compared with the plasma, kidney, lung, spleen or testicular concentration. This is consistent with unequal tissue distribution (Xue *etal.*, 2012). In rats, the primary phase I metabolic pathway of TP is hydroxylation into mono-hydroxylated triptolides that can subsequently undergo phase II metabolism into glucuronides and sulfates (Du *etal.*, 2014; Li *etal.*, 2008). CYP3A was identified to be primarily responsible for hydroxylation and dexamethasone, a CYP3A inducer, increased TP metabolism in rat liver microsomes (Ye *etal.*, 2010) and reduced TP-induced hepatotoxic effects (Ye *etal.*, 2010). Conversely, a single dose of TP that caused only mild toxicity in wild-type mice resulted in severe toxicity and death in cytochrome P450 gene deleted mice (Xue *etal.*, 2011). These findings suggest that TP itself is toxic (not dependent on metabolism) and the higher degree of toxicity in the liver may be due to a higher TP distribution to this organ.

TP is a reactive electrophile containing 3 epoxide groups that can bind to cellular macromolecules (Attia, 2010). It has been reported that TP can covalently bind to a subunit of the transcription factor II human complex (TFIIH) and cause inhibition of its DNA-dependent ATPase activity, which leads to the inhibition of RNA polymerase II mediated transcription (Titov *etal.*, 2011). Another group confirmed that TP inhibited total RNA and mRNA *de novo* synthesis. Up to 98% of genes were down-regulated in a human nonsmall cell lung cancer line after exposure to TP. TP also depleted RPB1, the main RNA polymerase II subunit. These *invitro* data suggest that inhibition of RNA synthesis may explain the pharmacology and toxicology of TP (Vispe *etal.*, 2009).

In order to better understand drug-induced liver injury (DILI), new tools and models are needed. Zebrafish are a promising animal model for studying DILI with many advantages over rodents (Vliegenthart *etal.*, 2014b). Advantages include convenient drug delivery, high fecundity, lower financial costs, optical clarity of larvae allowing real-time imaging of toxicity and suitability for high-throughput screening that is not possible with other vertebrate systems (Lieschke and Currie, 2007; McGrath and Li, 2008; Zon and Peterson, 2005). Recently, we have established a DILI model in zebrafish using paracetamol as the toxic agent (Vliegenthart *etal.*, 2014a). The main difference between the mammalian and zebrafish liver is the structural organization of the liver tissue. Instead of having the large bile ducts, portal veins and hepatic arteries organized in portal tracts, these are randomly allocated throughout the liver parenchyma in the zebrafish. The tri-lobed liver of the zebrafish is similar to other mammals with regard to biological function. This includes processing of lipids, vitamins, proteins, carbohydrates and the synthesis of serum proteins (Menke *etal.*, 2011). The metabolic properties of zebrafish regarding xenobiotics, including drugs, also have many similarities with mammals (Vliegenthart *etal.*, 2014b).

In this study we applied novel imaging tools to characterize a new model of DILI due to TP in zebrafish larvae. We performed histological examination, selective plane illumination microscopy (SPIM) and time-lapse imaging by 2D microscopy to characterize the time course of liver injury. The transcriptional changes induced by TP were investigated by RNA-sequencing. The results were consistent with mRNA synthesis inhibition and identified nitric oxide production as a protective pathway in the injury pathogenesis.

## MATERIALS AND METHODS

### 

#### 

##### Fish lines and husbandry

Experiments were conducted in accordance with the United Kingdom Animals (Scientific Procedures) Act 1986 in a United Kingdom Home Office-approved establishment. Zebrafish (*Danio rerio*) were maintained at 28.5 °C, as previously described by Westerfield (Westerfield, 2007). Established lines used were WIK and Tg(*-2.8lfabp*:GFP)^as3^ (Her *etal.*, 2003), where GFP is green fluorescent protein.

##### Chemical exposure

The wild-type WIK line was used for all experiments apart from imaging. Unless otherwise stated larvae were maintained at 28.5 °C in 50 ml conditioned water (CW). Larvae were exposed to TP (National Institutes for Food and Drug Control, China, >98% pure) dissolved in CW at concentrations described in the Results section. For the experiments testing the effect of TP on survival, 30 larvae were treated per 50 ml dish for 48 h (3–5 days postfertilization [dpf]). The concentrations tested were 0 µM (3 dishes), 0.8 µM (3 dishes), 1.0 µM (3 dishes), 1.2 µM (3 dishes), 1.4 µM (2 dishes), 1.6 µM (2 dishes) and 2 µM (2 dishes). For the experiments testing the effect of TP on copies of microRNA-122 (miR-122) per larvae, 30 larvae were treated per 50 ml dish for 48 h (3–5 dpf). The concentrations tested were 0 µM (3 dishes), 0.2 µM (3 dishes), 0.4 µM (3 dishes) and 0.8 µM (3 dishes). For the effect of TP on histology 30 larvae were treated per 50 ml dish up to 48 h (3–5 dpf). The concentrations tested were 0 µM (3 dishes), 0.2 µM (3 dishes), 0.4 µM (3 dishes) and 0.8 µM (3 dishes), the number of fish of which successful histology scoring could be obtained per treatment are given in the Results section. For all imaging experiments larvae were treated for 48 h (3–5 dpf) with a concentration of 0, 0.4, 0.8, 1.2 or 1.6 µM in agarose conditions as indicated in the methods of each technique. For the sequencing experiment 5 dpf larvae (*N* = 30) were treated per 50 ml dish for 6 h with a TP concentration of 0 µM (8 dishes) or 1.6 µM (8 dishes). For the NOS inhibitor studies 3 dpf larvae (*N* = 30) were treated per 50 ml dish for 24 h. With either TP concentration of 0 µM, 1.0 µM, or 1.6 µM with or without NOS inhibitor.

Experiments determining the effect of nitric oxide synthase (NOS) inhibition on TP were blinded such that the investigator scoring larval mortality did not know the treatment groups. When necessary, larvae were anaesthetized with MS-222 (tricaine methanesulfonate—40 µg ml^−1^) dissolved in CW. Mortality was assessed in a blinded fashion with no response to stimuli as the measured endpoint.

For adult zebrafish experiments 6 fish (3 treatment and 3 control) aged 12 months were used. Fish were exposed for 10 h to triptolide (1.6 µM in conditioned water) or conditioned water alone. Then fish were fixed for histopathology. A veterinary pathologist, who was blinded to treatment allocation, assessed the liver sections.

##### Histology

Larvae were submerged in 10% formalin and left to fix for at least 24 h at 4 °C before processing. Larvae were prepared for histology as described by Sabaliauskas *etal.* (2006) resulting in agarose blocks containing zebrafish larvae. The agarose blocks were then embedded in paraffin blocks using a Thermo Electron Excelsior tissue processor (Thermo, UK). This was achieved by serial immersion in the following: 70% ethanol (1 h), 90% ethanol (1 h), absolute ethanol (1 h ×4), xylene (1 h ×2), wax (1.3 h ×3). The blocks were sectioned at 4 µm and placed on a glass slide that was then incubated at 52 °C for at least an hour. Tissue sections were then dewaxed and rehydrated using an autostainer (ST5010 Autostainer XL, Leica Microsystems, UK) through three×5-min cycles of xylene, two ×3-min cycles of 100% ethanol, one ×2-min cycles of 95% ethanol and one ×5-min wash of distilled H_2_O (dH_2_O) and were then left to stand in dH_2_O until the next procedure. After rehydration, for hematoxylin and eosin (H&E), slides were stained in the same autostainer by sequential immersion in hematoxylin (5 min), dH_2_O (5 min), Scott’s tap water (2 min), dH_2_O (5 min), and eosin (3 min). Stained slides were then dehydrated (dH_2_O wash (45 s), 70% ethanol (30 s), 95% ethanol (30 s ×2), 100% ethanol (1 min ×2), ethanol/xylene (1 min), and xylene (1 min ×3)), and cover slipped using Pertex Mounting Medium (CellPath Ltd, UK). Fish were sectioned sagittally along the midline to facilitate examination of the liver.

Histology was scored by an accredited (DiplECVP) veterinary pathologist (author JDP), who was blinded to the treatment groups. A semiquantitative scoring system was used to grade the pathological changes noted in these livers. Briefly, each feature of interest was ranked as follows: 0 = absent; 1 = 1%–25% of total area examined; 2 = 26%–75%; 3 = 76%–100%. The features of interest were hepatocyte vacuolation, hepatocyte swelling, hepatocyte disarray, and oncotic necrosis.

##### Imaging

Fluorescent microscopy and SPIM were performed with fluorescent liver reporter zebrafish Tg(*−2.8fabp10*:GFP)^as3^. Time-lapse recording of zebrafish embryos was performed using an EVOS FL Cell Imaging System (Life Science). Up to 5 larvae (72 dpf) were oriented in 700 µl agar (0.75%, wt/vol) containing 84 mg/l MS-222. Once the agar was set, 750 µl of CW with TP (concentration as indicated in Results section) and MS-122 (84 mg/l) was added on top of the agar and the time-lapse recording was started. The incubator was set to 27 °C and pictures were taken hourly. ImageJ software (National Institutes of Health, Bethesda) was used to determine maximum GFP intensity in each image.

##### Selective plane illumination microscopy

A custom built SPIM system was used. This was based on the design previously published (Taylor *etal.*, 2012). A Vortran VersaLase multiple wavelength system with 3 laser diodes (405, 488 and 561 nm) was coupled to the SPIM illumination arm using a single mode optical fibre. The appropriate laser power was set using Stradus VersaLase software, and a power meter (Thorlabs Inc, PM100D) was used to verify collimated beam power. The resulting beam was then focused onto the sample (10× 0.3 NA Nikon CFI Fluor water dipping objective) to produce a light sheet. Imaging of the sample was through a 12× 0.8NA Nikon CFI LWD Plan Fluor water dipping objective (N16LWD-PF) and the resulting beam sent to 3 QI-Click Mono CCD cameras (Q-Imaging Inc) via a series of dichoric filters CFP Em/BW 479/40 nm (MF479-40), GFP Em/BW 525/39 nm (MF525-39), TxRed Em/BW 630/69 nm (MF630-60) (Thorlabs Inc). The entire system was controlled through a written interface operating in the Python language.

##### SPIM image acquisition and analysis

Fish were age matched and selected from the same batch of eggs. Images were acquired from the same orientation at 1.5 µm intervals, at 3, 4, and 5 dpf. A Matlab script was written to determine liver volume. Files were automatically segmented based on signal intensity (kept consistent across groups), a mask applied and the signal intensity and area of the mask calculated. This was summed over the volume of the stack and the pixel size used to calculate the volume. Rendering of 3D SPIM image datasets was performed using Amira 3D for Life Sciences v5.5.0; imported fluorescence images were kept consistent throughout time points and treatments and an isosurface was created for liver volume and structure visualization.

##### RNA extraction

Total RNA was extracted from pooled zebrafish larvae (30 larvae per sample) for qPCR and RNA-sequencing. Larvae were fixed in Qiazol after which they were disrupted using a tissue disruptor. Subsequently, total RNA was extracted using the miRNeasy mini kit (Qiagen, Venlo, The Netherlands) eluted in 30 µl RNAse free water.

##### PCR analysis

For microRNAs 1 µg of total RNA from pooled larvae was reverse transcribed into cDNA using the miScript II RT Kit (Qiagen) following manufacturer’s instructions. The synthesized cDNA was 10-fold diluted and used for cDNA template in combination with the miScript SYBR Green PCR Kit (Qiagen) using the specific miScript assays (Qiagen). Absolute quantification of microRNA was achieved by generating a standard curve using synthetic target. Standard curves were generated by reverse transcribing known concentrations of miScript microRNA mimics (Qiagen) in 0.1× TE buffer spiked with 10 ng/µl Poly-C (Sigma-Aldirch, Gillingham, UK). The resulting cDNA was measured using serial dilutions on 3 different plates to demonstrate minimal variability. The final concentration was divided by the number of larvae used within the pooled sample to obtain the average microRNA copy number per larva.

For mRNA, 1 µg of total RNA from pooled larvae was reverse transcribed into cDNA using the QuantiTect Reverse Transcription Kit (Qiagen) following manufacturer’s instructions. The synthesized cDNA was 10-fold diluted and used for cDNA template in combination with the QuantiTect SYBR Green PCR Kit (Qiagen) using the specific QuantiTect primer assays (Qiagen). Real-time PCR was performed on a Light Cycler 480 (Roche, Basel, Switzerland) using the recommended cycling parameters.

##### RNA-sequencing

For this experiment 5 dpf wild type (WIK) zebrafish were used and exposed to triptolide or vehicle control (DMSO). Each treatment group had 8 biological replicates, each replicate consisted of 30 pooled fish. For quality control RIN values were measured on the Agilent Technologies 2100 Bioanalyzer using the Eukaryote Total RNA Nano kit. All samples had a RIN of > 9.5. RNA Sequencing was performed using the Illumina NextSeq 550 system with 2 ×75 bp paired end runs. RNA libraries were prepared for each sample with the Truseq Stranded Total RNA Sample Prep LT Kit. Libraries were checked for size, purity and concentration with a high sensitivity DNA chip on the Agilent Technologies 2100 Bioanalyzer.

The raw sequences were quality assessed using FASTQC. Based on the output of the FASTQC analysis, the raw fastq sequences required no further preprocessing to remove contaminating primers and sequences were not collapsed within each sample.

The most recent Ensembl release (Rel84, March 2016) of zebrafish transcript sequences was downloaded using BioMart (Smedley *etal.*, 2015). Alignments (end-to-end, very-sensitive settings) to the reference set were performed using bowtie2 (Langmead and Salzberg, 2012). A requirement for concordant read pair mapping was applied, and all other alignments discarded. Alignments were stored in indexed BAM files. Raw “tag counts” (ie, sequences aligning) per sample were normalized to the sample with the lowest number of alignments, and counts converted to log2; “abundance normalized” data were not further quantile normalized for linear model fitting purposes.

##### Gene ontology enrichment analysis

Gene ontology (GO) and Kyoto encyclopedia of genes and genomes (KEGG) enrichment analysis for over or under representation of GO terms or KEGG pathways were done by using a hypergeometric test for likelihood due to chance, reporting anything more significant than the cut-off *P* value of .001.

##### Statistical analysis

Statistical differences in copies of microRNA per fish, histology scores, liver volume, difference in gene expression obtained by PCR and death rate were performed using Graphpad Prism (GraphPad Software, La Jolla, California). For RNA-seq pairwise comparisons of the 2 sample groups were performed on the normalized tag counts using linear modeling with the Bioconductor *limma* package (Ritchie *etal.*, 2015). Nominal statistical significance was set at *P* < .05, unless an adjusted *P* value was used (as described with the Results section).

## RESULTS

### 

#### Triptolide-Induced Liver Toxicity in Zebrafish Larvae

**Figure 1:**
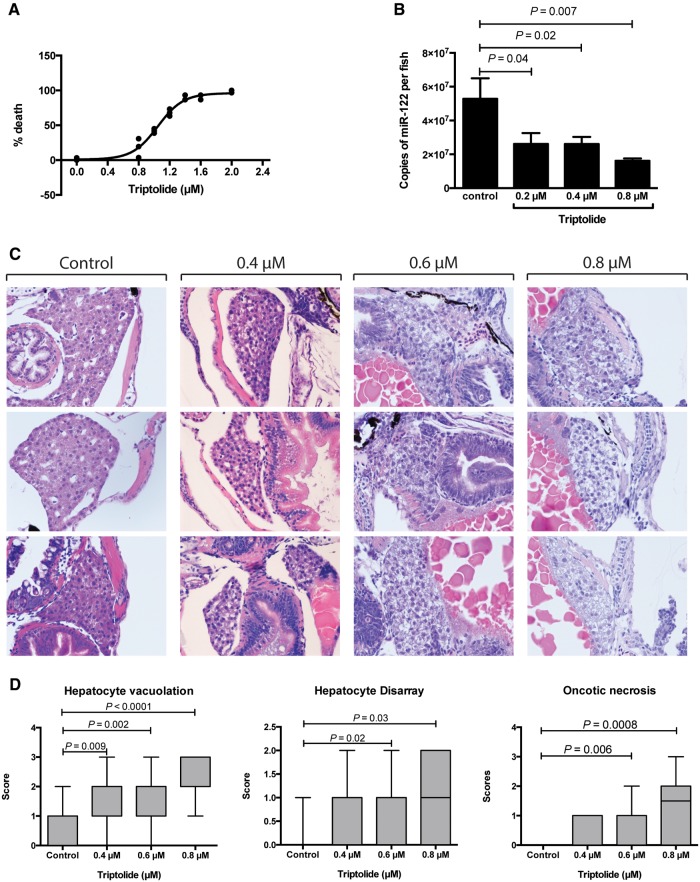
Effect of triptolide on zebrafish larvae after 48 h (3–5 dpf) exposure. A, Survival of zebrafish larvae after TP exposure at the concentrations indicated. Each dot represents mean mortality of 30 larvae. B, Copies of miR-122 per larva after 48 h of exposure at the concentrations indicated (from 3 to 5 dpf). C, Histological images of zebrafish larvae after exposures of the TP concentrations indicated. Three representative fish are presented per TP dose. D, Box plots (min to max) of histology scores for hepatocyte vacuolation, hepatocyte disarray and oncotic necrosis after TP exposure at the concentrations indicated (control *N* = 12, 0.4 µM *N* = 20, 0.6 µM =18 and 0.8 µM *N* = 18).

First, we determined whether TP induced liver injury in zebrafish. Larvae were exposed to TP for 48 h from 3 to 5 dpf. This exposure caused mortality with a dose-response relationship (Figure 1A). Histological examination of larvae revealed injury specifically to the liver. There was a dose response relationship between TP and hepatocyte vacuolation, oncotic necrosis and hepatocyte disarray subsequent to cell death (Figs. 1C and D and Supplementary Figure 1). These histological features were diffusely present throughout the liver tissue. Out of 38 zebrafish larvae treated with TP (0.8 µM) only 3 did not have histological evidence of liver injury. There was no discernible histological injury to other organs. To confirm that TP is hepatotoxic in adult zebrafish we exposed fish aged 12 months to TP (1.6 µM) or vehicle control for 10 h. There was hepatocyte necrosis in TP exposed adult zebrafish (Supplementary Figure 2). 

**Figure 2:**
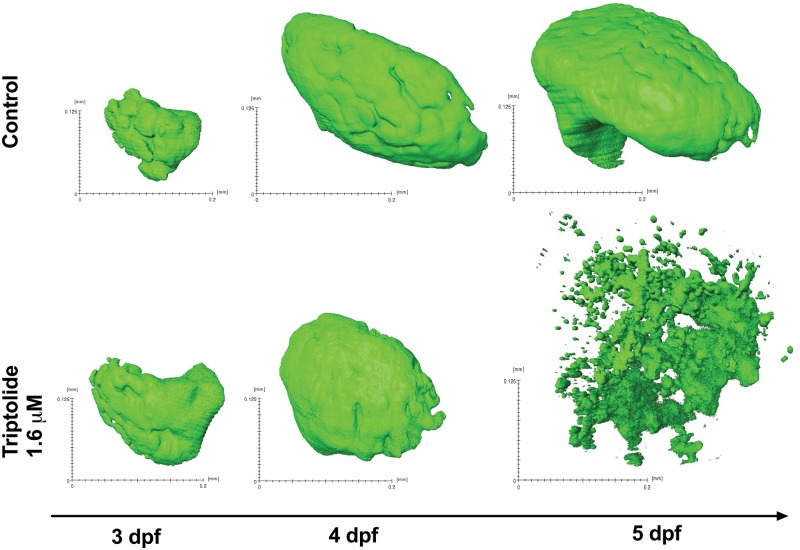
3D images of livers captured by SPIM. 3 dpf zebrafish larvae were exposed to 1.6 µM triptolide or vehicle control. After 6 h of exposure the first image was captured and subsequent images were taken at 4 and 5 dpf.

Our previous work has demonstrated that zebrafish release the liver specific microRNA, miR-122, from injured hepatocytes (Vliegenthart *etal.*, 2014a). TP exposure resulted in a significant decrease in the number of copies of miR-122 per larva (Figure 1B). Building on these data, SPIM was used to quantify liver volume in the zebrafish line Tg(*-2.8lfabp*:GFP)^as3^ (Figure 2). Images were captured for vehicle control (*N* = 10) and TP-treated fish (*N* = 6) at 5 dpf after 54 h of drug/vehicle exposure. Control livers increased in size from 3 to 5 dpf and, at 5 dpf, had a higher mean liver volume of 85.4 (SD 0.6) mm^3^ compared with no fluorescent signal for TP, *P* = .0002 (Figure 2). 

#### Onset of TP-Induced Hepatotoxicity Could Be Determined by Time-Lapse Microscopy

**Figure 3:**
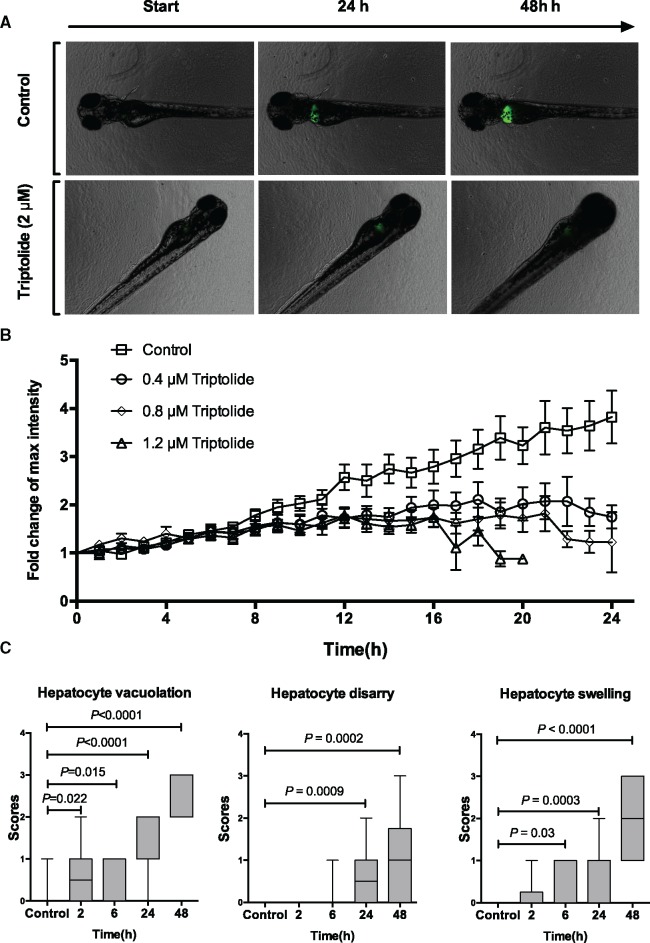
Time-course of triptolide-induced liver injury. A, Fluorescent images of control and TP exposed fish obtained during time-lapse experiments at the indicated time from start of exposure. B, Relative fold change of fluorescent intensity from baseline during TP exposure with the doses indicated (*N* = 15 larvae for each dose). C, Box plots (min to max) of histology scores of hepatocyte vacuolation, hepatocyte disarray and hepatocyte swelling during exposure to TP (0.8 µM) for the time durations indicated (control *N* = 17, 2 h *N* = 10, 6 h *N* = 9, 24 h *N* = 16, 48 h *N* = 20).

Time-lapse microscopy was used to characterize the time course of injury and identify an early time point for RNA sequencing. The fish line Tg*(-2.8lfabp*:GFP)^as3^ enabled quantification of the fluorescence intensity of the liver during TP exposure (Figure 3A). After 6 h, the fluorescent intensity of TP-treated fish started to decrease compared with control fish, suggesting that this time may represent the onset of hepatocyte injury (Figure 3B). Histological examination supported the data from time-lapse microscopy. There was statistically significant hepatocyte vacuolation and swelling 6-h postexposure to TP (Figure 3C). 

#### RNA-Seq Revealed Pathways Involved in Triptolide Exposure

RNA-seq was performed on 5 dpf larvae after exposure to TP for 6 h, an early time-point identified by microscopy and histology as being before the onset of fulminant hepatocyte death. A total of 16 sequencing experiments were performed (control *N* = 8 and treatment *N* = 8). Each replicate consisted of 30 pooled larvae, therefore 480 larvae were included in total.

**Figure 4:**
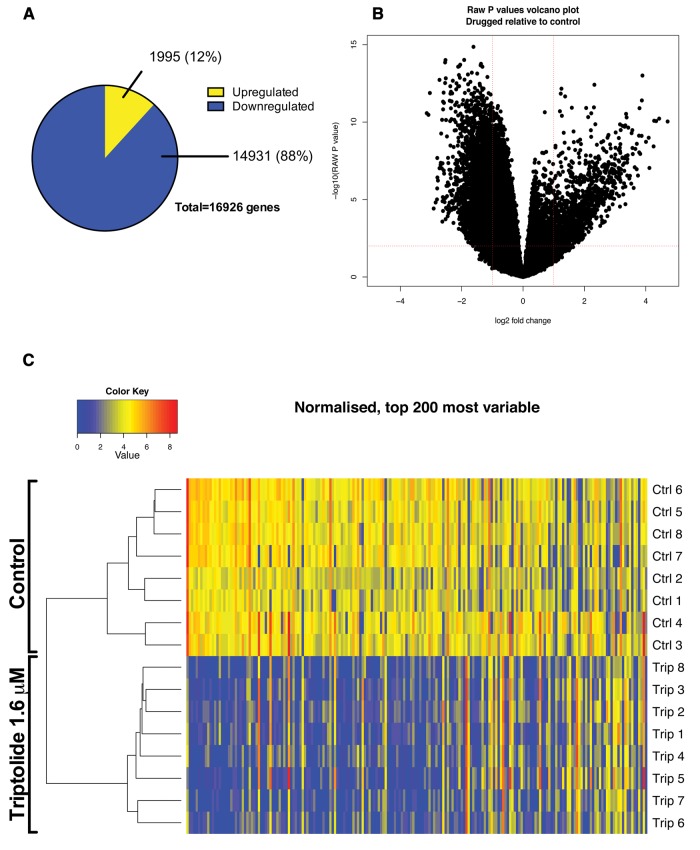
RNA-sequencing of zebrafish larvae exposed to triptolide. RNA-seq was performed on 5 dpf larvae after exposure to triptolide (1.6 µM) for 6 h. A total of 16 sequencing experiments were performed (control *N* = 8 and treatment *N* = 8). Each individual experiment consisted of 30 pooled larvae, therefore 480 larvae were included in total. A, Proportion of down-regulated and up-regulated genes. B, Volcano plot of adjusted *P* value *versus* log2 fold change of triptolide treated fish *versus* control. C, Clustered heatmap with the top 200 most variable genes.

A total of 57 264 transcripts were identified of which 16 926 were statistically significantly differentially expressed with an adjusted *P* value of 0.01 or less. Of the differentially expressed transcripts, 1995 were up-regulated and 14 931 down-regulated. Of these significant transcripts, 1433 (12%) were more than 2-fold up-regulated and 4675 (88%) were more than 2-fold down-regulated (Figure 4A). Unsupervised clustering separated the control group from the TP exposed group (Figure 4B). The top 10 most up- and down-regulated genes based on fold change are presented in Table 1.

**Table 1:** Top 10 Most Up-Regulated and Down-Regulated Genes

ID	Symbol	Fold Change	Adjusted *P* Value
ENSDART00000128835	NA	26.2	2.39×10^−8^
ENSDART00000154981	wu:fi47d06	21.7	1.80×10^−8^
ENSDART00000103858	LOC557782	20.3	2.29×10^−8^
ENSDART00000025847	TNF-α	19.3	2.24×10^−8^
ENSDART00000037557	admp	19.0	3.16×10^−7^
ENSDART00000062715	NA	17.4	3.17×10^−06^
ENSDART00000157090	LOC557782	17.0	1.30×10^−7^
ENSDART00000129697	ifnphi3	16.1	1.97×10^−7^
ENSDART00000146927	LOC100330560	14.8	3.74×10^−10^
ENSDART00000019296	gdf9	14.6	3.33×10^−09^
ENSDART00000145976	hsp47	−6.8	1.28×10^−05^
ENSDART00000138941	shox2	−6.9	9.08×10^−06^
ENSDART00000048182	v2rh32	−7.0	1.07×10^−06^
ENSDART00000134215	grik1a	−7.0	3.09×10^−05^
ENSDART00000169378	mmp13a	−7.0	0.0001
ENSDART00000101289	zgc:153395	−7.2	0.0002
ENSDART00000054588	spon2b	−7.5	0.0003
ENSDART00000132490	NA	−8.2	1.65×10^−09^
ENSDART00000082333	LOC792903	−8.5	1.22×10^−8^
ENSDART00000078154	npas4	−8.8	1.03×10^−8^

RNA-sequencing of zebrafish larvae exposed to triptolide (1.6 µM) for 6 h compared with vehicle control.

Gene ontology (GO) enrichment analysis revealed 32 significantly enriched terms in biological process, 16 enriched terms in cellular component and 17 enriched terms in molecular function (Supplementary Table 1). The most significant enriched GO terms were translation (*P *= 4.35×10^−51^) in the biological process ontology, structure constituent of ribosome (*P *= 3.13×10^−64^) in the molecular function ontology and ribosome (*P *= 9.09×10^−61^) in the cellular component ontology. KEGG pathway analysis revealed 6 up-regulated pathways of which ribosome (*P *= 1.87×10^−72^) and cardiac muscle (*P* = 8.01×10^−7^) pathways were the most significant. Another 18 down-regulated KEGG pathways were identified, of which spliceosome (*P *= 4.19×10^−5^) and notch signaling (*P *= .0008) were the most significant (Table 2).

**Table 2:** KEGG Pathways

Up/Down (trip vs Ctrl)	Pathway Description	*P* Value
Up	Ribosome	1.87E-86
Up	Cardiac muscle contraction	8.01E-07
Up	Oxidative phosphorylation	0.002544
Up	Steroid hormone biosynthesis	0.006209
Down	Spliceosome	4.19E-08
Down	Notch signaling pathway	0.000845
Down	Progesterone-mediated oocyte maturation	0.004103
Down	Ubiquitin mediated proteolysis	0.005622
Down	Gap junction	0.00651

RNA-sequencing of zebrafish larvae exposed to triptolide (1.6 µM) for 6 h compared with vehicle control.

**Figure 5:**
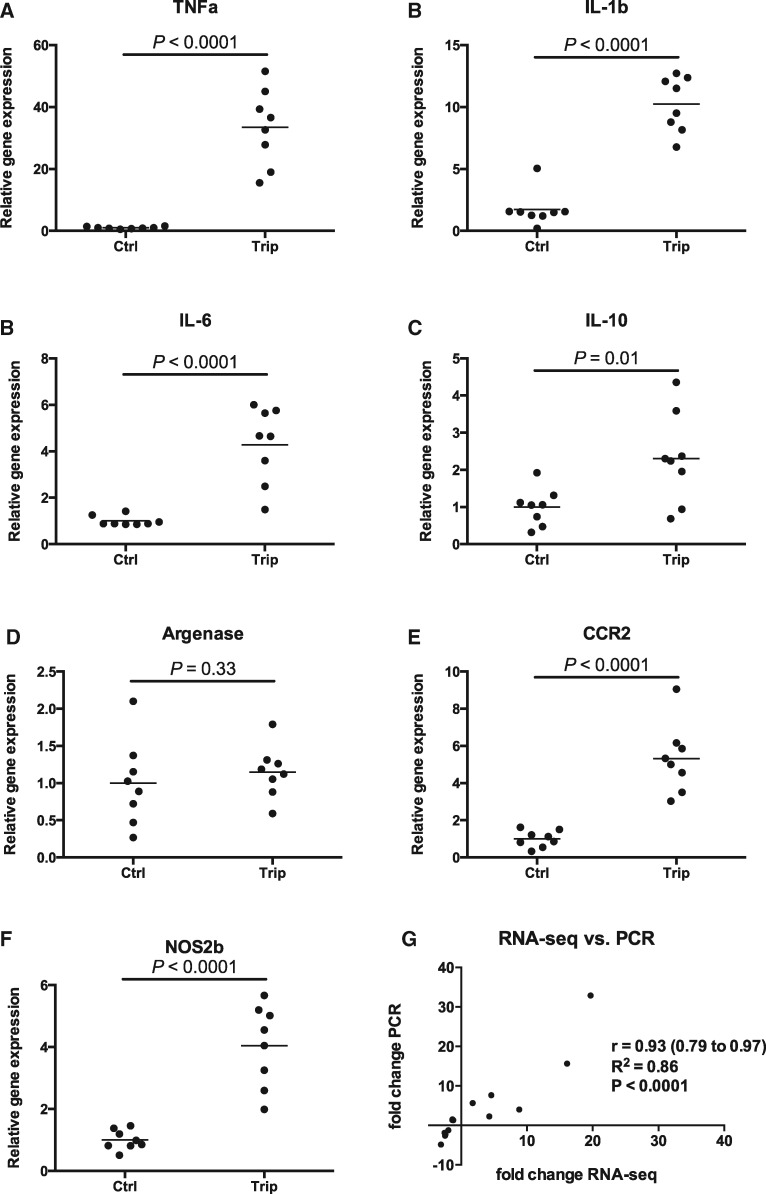
Inflammatory gene expression in zebrafish larvae treated with triptolide (1.6 µM) for 6 h. A–F, Relative gene expression of various inflammatory associated genes measured by PCR (*N* = 8 pooled sample of 30 larvae for each group, each dot represents one pooled sample and line represents mean). G, correlation between fold change of 14 genes obtained by RNA-seq *versus* PCR. All genes measured by PCR were normalized by MRPS18B.

Multiple inflammation associated genes were up-regulated and validated by qPCR. Nitric oxide synthase 2b (NOS2b) was 4.0-fold increased (*P *<* *.0001) in the TP treated fish compared with control along with other genes involved in an inflammatory response including TNF-α (32.9-fold, *P *<* *.0001), IL-1b (7.6-fold, *P* < .0001), IL-6 (4.0-fold, *P* < .0001), IL-10 (2.3-fold, *P* = .01) and CCR2 (5.6-fold, *P* < .0001) (Figs. 5A–E). 

To further validate the sequencing results, 14 transcripts chosen from differentially regulated pathways and genes involved in the immune response were measured by qPCR. The fold change obtained by RNA-sequencing correlated with the fold change measured by qPCR with a Pearson’s *r* of 0.93, *P* < .0001 (Figure 5G).

#### Nitric Oxide Synthase Is Involved in Triptolide Toxicity

**Figure 6:**
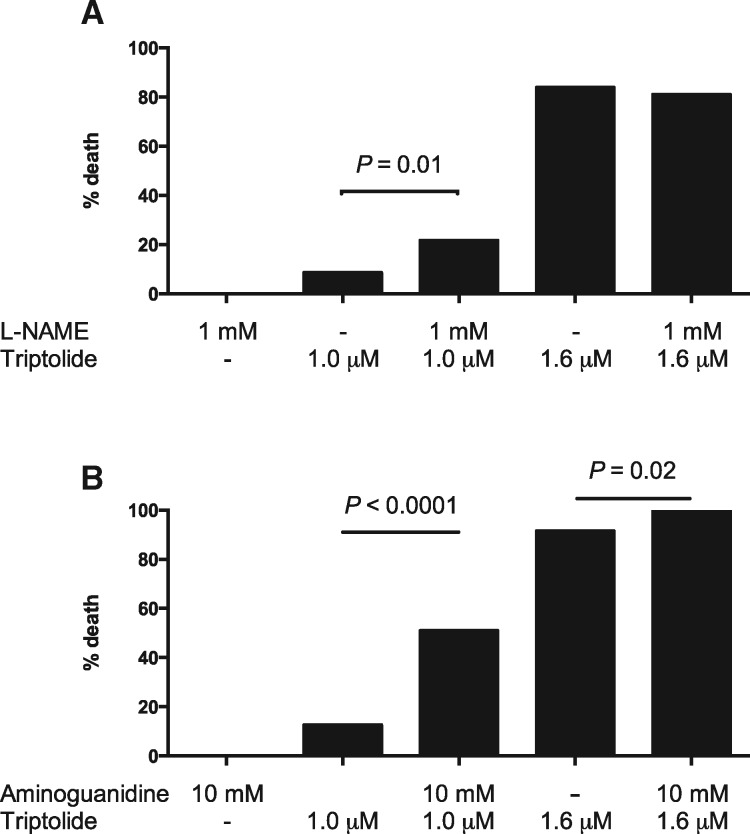
Effect on mortality of co-treatment with (A) L-NAME or (B) aminoguanidine. Three-dpf old larvae were exposed for 24 h to triptolide and/or the NOS inhibitor indicated. Each dose was tested on at least 60 larvae. Mortality was assessed blinded to the treatment groups. Fisher’s exact test was used for statistical differences between groups.

Finally, in order to explore a mechanistic role for NOS2b in TP-induced toxicity, the effect of the nonselective NOS inhibitor L-NAME and the selective inducible NOS (iNOS) inhibitor aminoguanidine was determined. Neither compound caused any effect on zebrafish survival when applied alone. Co-treatment with TP and either L-NAME or aminoguanidine significantly increased larval mortality (Figure 6). 

## DISCUSSION

TP, the primary active compound in *Tripterygium wilfordii*, has a long history of use because of its purported neuroprotective, anti-inflammatory and contraceptive effects (Chen, 2001; Liu, 2011; Zheng *etal.*, 2013; Ziaei and Halaby, 2016). Due to its high incidence of hepatotoxicity, its utility in clinical medicine is limited (Li *etal.*, 2014).

Zebrafish larvae are as proliferative and easily accessible as *invitro* cell culture models while providing *invivo* complexity comparable to larger vertebrate models (Vliegenthart *etal.*, 2014b). In addition, zebrafish have substantially lower cost and the larvae are optically clear so are suitable for high throughput screening (Lieschke and Currie, 2007; McGrath and Li, 2008; Zon and Peterson, 2005). This unique combination of characteristics makes the zebrafish an attractive model species for studying DILI.

To investigate whether zebrafish larvae could be used as a tool to study TP-induced DILI, larvae were exposed to increasing concentrations of TP. Larvae died with a dose-response relationship in the micromolar range and histological examination revealed that TP induced highly reproducible hepatic necrosis without affecting other organs. The plasma concentration of triptolide in humans (*C*max) is reported to be around 0.15–0.4 µM.(Yao *etal.*, 2006) In rats, a plasma concentration of 1.5µM has been reported to be associated with histological liver cell necrosis (Shao *etal.*, 2007). These human and rodent studies are consistent with the concentrations that produce hepatotoxicity in our zebrafish larvae, which supports the translational relevance of our fish model. Ours is the first study confirming that TP-induced hepatotoxicity can be modeled in zebrafish larvae. The administration of TP is straightforward and the resultant liver injury is reproducible and tractable. This is in contrast to paracetamol, an archetypal compound used to induce hepatocyte necrosis, which is variable with regard to histological liver injury in larvae and requires millimolar water concentrations for an effect (Vliegenthart *etal.*, 2014a). The enhanced ability of TP to induce liver toxicity is likely due to a combination of its pharmacokinetics and its ability to induce injury without need for metabolism. We believe that the data from these studies with triptolide confirm the benefits of zebrafish as a model organism to screen for DILI. We acknowledge, however, that there are limitations to our model and some important future questions to be addressed. When selecting an animal model for toxicity testing, characterization of the metabolic properties of the selected species is very important. TP is metabolized in rodents by the cytochrome P450 system. These enzymes have been demonstrated to be present and active in zebrafish embryos (Goldstone *etal.*, 2010). However, further experiments are required to confirm that this system is responsible for TP metabolism in our zebrafish larvae model. We demonstrate liver injury in adult zebrafish at a concentration of TP that is toxic in larvae. This demonstrates that TP hepatotoxicity is not an idiosyncrasy of larvae. It is probable that the sensitivity of the larvae to TP will be different to adult fish, for example, because of differences in tissue penetration of drug and immaturity of drug metabolism. Furthermore, while the adult and larvae both develop liver cell necrosis at the same concentration of TP, this is not confirmation that the same mechanisms underlie toxicity across the zebrafish life span.

We previously reported that miR-122 increases in the circulation of patients with DILI with superior sensitivity and specificity compared with the currently used clinical biomarker alanine aminotransferase (ALT) (Vliegenthart *etal.*, 2015). Using *in situ* hybridization, we reported that miR-122 is specifically expressed in the zebrafish liver and is released into the circulation with DILI. As in humans, in zebrafish miR-122 can be used as a more sensitive and specific biomarker than ALT (Vliegenthart *etal.*, 2014a). The present study reports that the number of copies miR-122 per zebrafish larvae is decreased with TP-induced DILI, indicating that miR-122 can also be utilized as a biomarker for DILI in whole zebrafish larvae.

By exploiting the optical transparency of the zebrafish larvae in combination with the liver specific fluorescent reporter transgenic fish line, Tg(*-2.8lfabp*:GFP)^as3^, we were able to capture 3D images by using SPIM of the fish liver during TP-induced DILI. This confirmed that the liver volume (as reported by fluorescence) is substantially reduced with injury. Because of low bleaching, high acquisition speed and high depth penetration, SPIM is well suited for imaging intact fully functioning zebrafish larvae. These characteristics make SPIM suited for time-lapse imaging of biological processes over long periods of time (Huisken and Stainier, 2009; Weber and Huisken, 2011). This is potentially valuable in studies of development (Kaufmann *etal.*, 2012), functional imaging of multiple brain regions (Panier *etal.*, 2013) and heart function (Mickoleit *etal.*, 2014). This is the first study confirming that SPIM has sufficient depth penetration to image the zebrafish liver from 3 to 5 dpf. SPIM is a tool that could be further exploited in the field of studying liver development and injury in zebrafish.

Fluorescent time-lapse microscopy allowed us to characterize the time course of injury and identified an early time point for RNA sequencing, which demonstrated that TP has similar effects on RNA transcription as *invitro* models. TP covalently binds to a subunit of TFIIH that leads to inhibition of RNA polymerase II transcription initiation (Titov *etal.*, 2011). RNA expression studies using microarrays have shown that both total RNA and mRNA *de novo* synthesis were lowered in a TP treated cancer cell line compared with control. Among the down-regulated genes was RPB1, the main RNA polymerase II subunit (Vispe *etal.*, 2009). These *invitro* data indicate that inhibition of RNA synthesis could explain TP hepatotoxicity. In our study, the RNA-sequencing data also demonstrated that TP leads to a general down-regulation of gene expression, with 88% being down-regulated in TP treated larvae compared with vehicle control. Notably, this effect on gene expression was measured at an early time-point before fulminant hepatic necrosis had started. Therefore, we propose that inhibition of RNA synthesis may be the mechanism that causes liver toxicity. In line with this hypothesis, gene ontology analysis revealed that, in general, GO terms involved in RNA transcription, such as nucleic acid metabolic processes, transcription, regulation of gene expression and RNA biosynthesis, were significantly reduced. Also KEGG pathways involved in RNA transcription went down, including spliceosome and RNA polymerase. GO terms in biological processes involved in protein synthesis—such as translation, cellular protein metabolic process and protein metabolic process—were increased. We speculate that up-regulation of processes involved in protein synthesis might be a response to lower template mRNA; an attempt by the organism to maintain equilibrium.

Other up-regulated GO terms were involved in the immune response, defense response, response to other organisms, immune system process, response to viruses and response to lipopolysaccharide. PCR confirmed that multiple inflammatory markers were increased in the TP-treated fish compared with control, with the cytokine TNF-α being increased 32.9-fold. This suggests that, besides a general down-regulation of transcription, inflammation potentially has a role in TP-induced DILI. In DILI, the liver injury caused by the drug or its metabolites is often an initiating event for an immune response which determines the extent of liver injury (Ju and Reilly, 2012). Liver necrosis with inflammatory cell infiltration has been reported in TP-induced hepatotoxicity (Fu *etal.*, 2011) and Th17/Treg imbalance has been associated with the exacerbation of liver inflammation in TP-induced hepatotoxicity (Wang *etal.*, 2014). We speculate the increase in inflammatory genes in our larval model reflects activation of circulating innate immune cells. Unlike liver cells, these circulating cells may be able to up-regulate their inflammatory genes because of a lower intra-cellular TP concentration. This is speculative and requires confirmation in future studies.

Three NOS isoforms have been identified in humans: a neuronal NOS1, an inducible NOS2 and an endothelial NOS3 enzyme. One NOS1 and 2 NOS2 (NOS2A and NOS2B) genes have been reported to be present in the zebrafish genome. Both liposaccharide stimulation and tail cut injury induces the NOS2 isoforms in zebrafish (Lepiller *etal.*, 2009). Knockdown of NOS2b reduced leukocyte attraction to ventral fin wounds (Wittmann *etal.*, 2015). These data indicate that NOS2b is involved in inflammation. Our results suggest that inhibition of NOS2b in our model increased TP toxicity. This is consistent with a protective role for NOS2b. The protective role of NOS has also been reported in a model of paracetamol liver injury in mice (Hinson *etal.*, 2002). Various other models have also demonstrated that NO protects the liver against oxidative stress induced by ethanol, (Nanji *etal.*, 1995), H_2_O_2_ (Kim *etal.*, 1995) and CCl_4_ (Zhu and Fung, 2000). Future research should identify compounds that can limit TP toxicity once it is established and promote liver regeneration, as such compounds could be clinically useful.

In conclusion, TP induces hepatic necrosis in zebrafish larvae and inhibits RNA synthesis in line with previous published data. The hepatocyte necrosis induces an inflammatory response, which partially determines outcome. TP is a model compound for inducing DILI in zebrafish. Zebrafish have experimental properties which complement rodent models of toxicity.

## SUPPLEMENTARY DATA

Supplementary data are available at *Toxicological Sciences* online.

## FUNDING

NC3Rs PhD Studentship (NC/K001485/1 to A.D.B.V.); NHS Research Scotland (NRS) through NHS Lothian and a BHF Centre of Research Excellence Award (to J.W.D).

## Supplementary Material

Supplementary Data
