# Supplementary material for: Characterization of Triptolide-Induced Hepatotoxicity by Imaging and Transcriptomics in a Novel Zebrafish Model
Source: Toxicol Sci. 2017 Jul 20;159(2):380–91. doi: 10.1093/toxsci/kfx144 (PMC5837554; doi:10.1093/toxsci/kfx144)
Supplement: Supplementary Data [file kfx144_supp.docx]

**Characterization of triptolide-induced hepatotoxicity by imaging and transcriptomics in a novel zebrafish model**

AD Bastiaan Vliegenthart, Chunmin Wei, Charlotte Buckley, Cécile Berends, Carmelita MJ de Potter, Sarah Schneemann, Jorge Del Pozo, Carl Tucker, John J Mullins, David J Webb, James W Dear

| **Supplementary Figure 1** | Page 2 |
| --- | --- |
| **Supplementary Figure 2** | Page 3 |
| **Supplementary Table 1** | Page 4-8 |


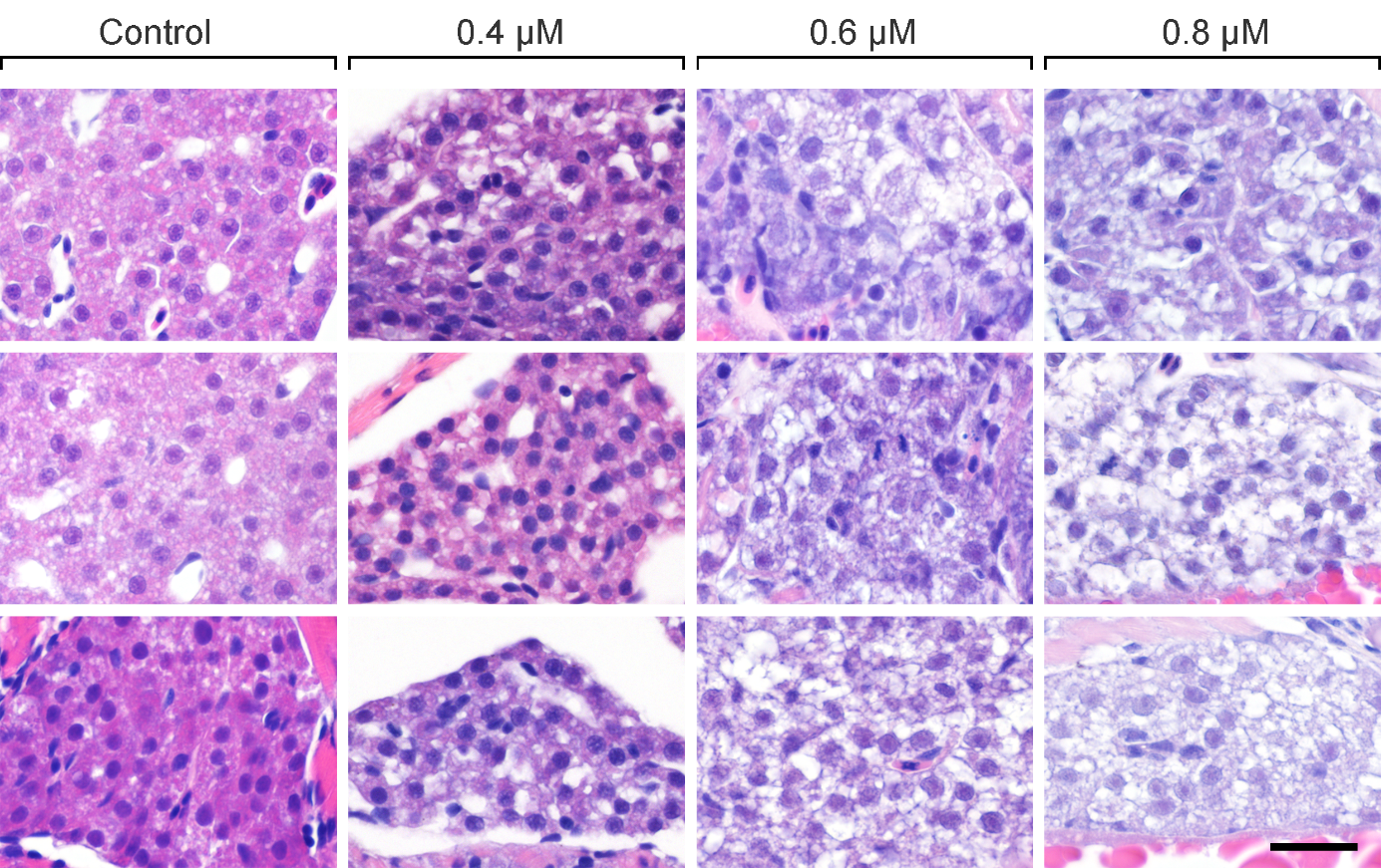


C

C

C

B

B

B

A

A

A

Supplementary Figure 1. Effect of triptolide on zebrafish larvae after 48 hours (3-5dpf) exposure. Magnified histological images at x400 of zebrafish larvae liver after water exposure to triptolide at the concentrations indicated. Three representative fish are presented for each dose. Note the presence of dose dependent increase in hepatocyte vacuolation (A), necrosis (with nuclei breaking down (B) and karyolysis (C)) and the presence of disarray (red lines). The latter two features are absent from the control group. Scale bar = 20µm.


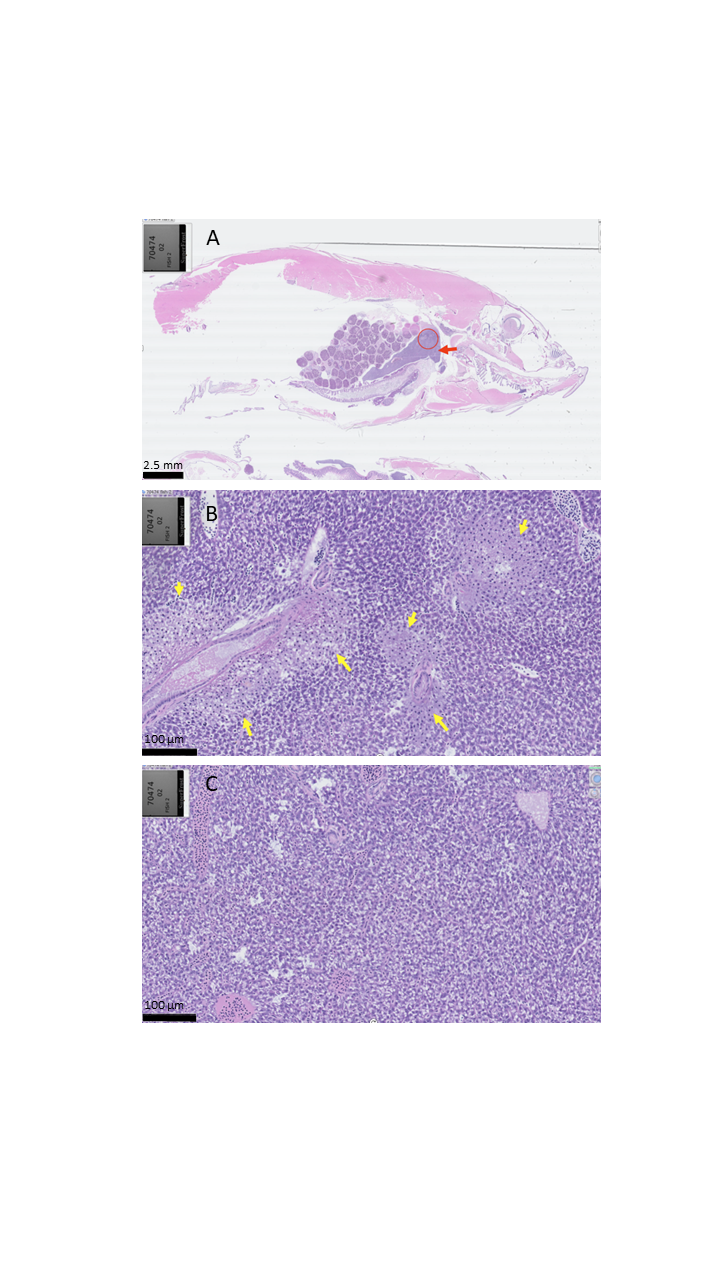


Supplementary Figure 2. Histological images of adult zebrafish after exposure to triptolide (1.6 μM) for 10 hours. (A) Subgross view. Liver is indicated by the red arrow. The area displaying hepatic necrosis is indicated by the circle. (B) Arrows denote lesions consistent with hepatic necrosis after triptolide exposure. (C) Control adult zebrafish after exposure to vehicle control. Normal liver, with no areas of necrosis.

Supplementary Table 1. Significantly enriched GO terms in response to triptolide in zebrafish larvae (with *P* < 0.001 cutoff).

|  | GO Term | Up/Down | PValux10 |
| --- | --- | --- | --- |
| Biological Process | translation | Up | 4.35x10^-51^ |
|  | cellular protein metabolic process | Up | 2.31x10^-13^ |
|  | protein metabolic process | Up | 1.56x10^-12^ |
|  | cellular biosynthetic process | Up | 7.93x10^-12^ |
|  | biosynthetic process | Up | 8.50x10^-12^ |
|  | macromolecule biosynthetic process | Up | 4.12x10^-10^ |
|  | cellular macromolecule biosynthetic process | Up | 5.81x10^-10^ |
|  | gene expression | Up | 5.19x10^-08^ |
|  | generation of precursor metabolites and energy | Up | 5.81x10^-07^ |
|  | immune response | Up | 2.35x10^-06^ |
|  | regulation of cell cycle | Up | 2.41x10^-06^ |
|  | translational elongation | Up | 2.58x10^-06^ |
|  | defense response | Up | 9.92x10^-06^ |
|  | primary metabolic process | Up | 1.24x10^-05^ |
|  | response to other organism | Up | 1.83x10^-05^ |
|  | multi-organism process | Up | 2.69x10^-05^ |
|  | immune system process | Up | 5.92x10^-05^ |
|  | cellular metabolic process | Up | 9.07x10^-05^ |
|  | response to biotic stimulus | Up | 0.0001 |
|  | embryo development | Up | 0.0002 |
|  | carbohydrate catabolic process | Up | 0.0002 |
|  | hormone biosynthetic process | Up | 0.0003 |
|  | energy derivation by oxidation of organic compounds | Up | 0.0004 |
|  | metabolic process | Up | 0.0005 |
|  | glycolysis | Up | 0.0005 |
|  | glucose metabolic process | Up | 0.0006 |
|  | cellular carbohydrate catabolic process | Up | 0.0006 |
|  | alcohol catabolic process | Up | 0.0006 |
|  | respiratory electron transport chain | Up | 0.0006 |
|  | purine nucleotide biosynthetic process | Up | 0.0006 |
|  | response to virus | Up | 0.0009 |
|  | response to lipopolysaccharide | Up | 0.0009 |
|  | nucleic acid metabolic process | Down | 4.83x10^-27^ |
|  | cellular nitrogen compound metabolic process | Down | 2.61x10^-23^ |
|  | nucleobase, nucleoside, nucleotide and nucleic acid metabolic process | Down | 3.95x10^-23^ |
|  | transcription | Down | 4.01x10^-23^ |
|  | regulation of transcription | Down | 2.65x10^-22^ |
|  | nitrogen compound metabolic process | Down | 4.84x10^-22^ |
|  | regulation of nucleobase, nucleoside, nucleotide and nucleic acid metabolic process | Down | 3.00x10^-21^ |
|  | regulation of gene expression | Down | 4.36x10^-21^ |
|  | regulation of nitrogen compound metabolic process | Down | 5.67x10^-21^ |
|  | regulation of macromolecule biosynthetic process | Down | 1.24x10^-20^ |
|  | regulation of biosynthetic process | Down | 1.35x10^-20^ |
|  | regulation of cellular biosynthetic process | Down | 1.80x10^-20^ |
|  | regulation of cellular metabolic process | Down | 1.18x10^-19^ |
|  | RNA metabolic process | Down | 5.84x10^-19^ |
|  | regulation of primary metabolic process | Down | 4.89x10^-18^ |
|  | regulation of macromolecule metabolic process | Down | 6.20x10^-18^ |
|  | gene expression | Down | 8.92x10^-18^ |
|  | regulation of metabolic process | Down | 1.99x10^-17^ |
|  | cellular macromolecule metabolic process | Down | 6.59x10^-14^ |
|  | regulation of RNA metabolic process | Down | 6.65x10^-12^ |
|  | regulation of transcription, DNA-dependent | Down | 8.54x10^-12^ |
|  | transcription, DNA-dependent | Down | 1.22x10^-11^ |
|  | nervous system development | Down | 1.52x10^-11^ |
|  | multicellular organismal development | Down | 1.82x10^-11^ |
|  | system development | Down | 2.66x10^-11^ |
|  | RNA biosynthetic process | Down | 2.68x10^-11^ |
|  | RNA processing | Down | 8.40x10^-11^ |
|  | multicellular organismal process | Down | 2.38x10^-10^ |
|  | developmental process | Down | 3.65x10^-10^ |
|  | macromolecule metabolic process | Down | 1.51x10^-09^ |
|  | regulation of cellular process | Down | 1.76x10^-09^ |
|  | anatomical structure development | Down | 2.51x10^-09^ |
|  | regulation of biological process | Down | 1.07x10^-08^ |
|  | cellular macromolecule biosynthetic process | Down | 2.01x10^-08^ |
|  | brain development | Down | 4.16x10^-08^ |
|  | organ development | Down | 4.46x10^-08^ |
|  | central nervous system development | Down | 5.35x10^-08^ |
|  | macromolecule biosynthetic process | Down | 5.73x10^-08^ |
|  | neurogenesis | Down | 2.13x10^-07^ |
|  | cellular metabolic process | Down | 5.09x10^-07^ |
|  | generation of neurons | Down | 7.20x10^-07^ |
|  | biological regulation | Down | 1.09x10^-06^ |
|  | skeletal system development | Down | 1.15x10^-06^ |
|  | forebrain development | Down | 1.23x10^-06^ |
|  | neuron differentiation | Down | 1.32x10^-06^ |
|  | cellular developmental process | Down | 2.33x10^-06^ |
|  | cartilage development | Down | 2.83x10^-06^ |
|  | embryo development ending in birth or egg hatching | Down | 3.57x10^-06^ |
|  | chordate embryonic development | Down | 3.57x10^-06^ |
|  | cellular biosynthetic process | Down | 8.45x10^-06^ |
|  | cell fate commitment | Down | 9.71x10^-06^ |
|  | cell differentiation | Down | 1.36x10^-05^ |
|  | biosynthetic process | Down | 2.24x10^-05^ |
|  | cellular process | Down | 2.74x10^-05^ |
|  | cell fate specification | Down | 2.84x10^-05^ |
|  | mRNA processing | Down | 3.09x10^-05^ |
|  | sensory organ development | Down | 3.52x10^-05^ |
|  | ncRNA processing | Down | 4.28x10^-05^ |
|  | cellular component organization | Down | 4.66x10^-05^ |
|  | skeletal system morphogenesis | Down | 5.29x10^-05^ |
|  | ribosome biogenesis | Down | 8.59x10^-05^ |
|  | primary metabolic process | Down | 0.0001 |
|  | ribonucleoprotein complex biogenesis | Down | 0.0001 |
|  | embryonic skeletal system development | Down | 0.0001 |
|  | anatomical structure morphogenesis | Down | 0.0001 |
|  | regulation of signaling pathway | Down | 0.0001 |
|  | rRNA processing | Down | 0.0002 |
|  | ncRNA metabolic process | Down | 0.0002 |
|  | embryonic skeletal system morphogenesis | Down | 0.0002 |
|  | camera-type eye development | Down | 0.0002 |
|  | hindbrain development | Down | 0.0003 |
|  | eye development | Down | 0.0003 |
|  | mRNA metabolic process | Down | 0.0003 |
|  | neuron development | Down | 0.0003 |
|  | embryonic cranial skeleton morphogenesis | Down | 0.0004 |
|  | cellular response to stimulus | Down | 0.0004 |
|  | rRNA metabolic process | Down | 0.0004 |
|  | RNA modification | Down | 0.0005 |
|  | cellular membrane organization | Down | 0.0005 |
|  | membrane organization | Down | 0.0005 |
|  | embryonic organ morphogenesis | Down | 0.0005 |
|  | generation of neurons in the forebrain | Down | 0.0005 |
|  | forebrain neuron differentiation | Down | 0.0005 |
|  | chromatin organization | Down | 0.0006 |
|  | neural crest cell differentiation | Down | 0.0006 |
|  | anterior/posterior pattern formation | Down | 0.0007 |
|  | peroxisome organization | Down | 0.0008 |
|  | cardioblast differentiation | Down | 0.0008 |
|  | regionalization | Down | 0.0008 |
|  | RNA splicing | Down | 0.0009 |
|  | neural crest cell development | Down | 0.0009 |
|  | pattern specification process | Down | 0.0009 |
|  | chromosome organization | Down | 0.0009 |
| Molecular Function | structural constituent of ribosome | MF | 3.13x10^-64^ |
|  | structural molecule activity | MF | 2.09x10^-41^ |
|  | rRNA binding | MF | 6.47x10^-08^ |
|  | hydrogen ion transmembrane transporter activity | MF | 4.97x10^-06^ |
|  | actin binding | MF | 1.26x10^-05^ |
|  | monovalent inorganic cation transmembrane transporter activity | MF | 8.25x10^-05^ |
|  | inorganic cation transmembrane transporter activity | MF | 0.0002 |
|  | cytoskeletal protein binding | MF | 0.0002 |
|  | cytochrome-c oxidase activity | MF | 0.0004 |
|  | heme-copper terminal oxidase activity | MF | 0.0004 |
|  | oxidoreductase activity, acting on heme group of donors | Up | 0.0004 |
|  | oxidoreductase activity, acting on heme group of donors, oxygen as acceptor | Up | 0.0004 |
|  | cytokine receptor binding | Up | 0.0004 |
|  | cation transmembrane transporter activity | Up | 0.0004 |
|  | substrate-specific transmembrane transporter activity | Up | 0.0007 |
|  | ion transmembrane transporter activity | Up | 0.0009 |
|  | nucleic acid binding | Down | 7.69x10^-22^ |
|  | transcription regulator activity | Down | 1.36x10^-16^ |
|  | DNA binding | Down | 1.63x10^-14^ |
|  | sequence-specific DNA binding transcription factor activity | Down | 4.96x10^-13^ |
|  | sequence-specific DNA binding | Down | 1.28x10^-09^ |
|  | binding | Down | 1.71x10^-08^ |
|  | zinc ion binding | Down | 7.20x10^-06^ |
|  | ubiquitin thiolesterase activity | Down | 0.0002 |
|  | transferase activity, transferring phosphorus-containing groups | Down | 0.0005 |
|  | transition metal ion binding | Down | 0.0006 |
| Cellular Component | ribosome | Up | 9.09x10^-61^ |
|  | ribonucleoprotein complex | Up | 3.48x10^-46^ |
|  | non-membrane-bounded organelle | Up | 7.41x10^-33^ |
|  | intracellular non-membrane-bounded organelle | Up | 7.41x10^-33^ |
|  | macromolecular complex | Up | 1.67x10^-24^ |
|  | cytoplasmic part | Up | 4.73x10^-24^ |
|  | cytoplasm | Up | 1.01x10^-17^ |
|  | ribosomal subunit | Up | 1.08x10^-10^ |
|  | small ribosomal subunit | Up | 4.86x10^-08^ |
|  | intracellular part | Up | 6.25x10^-08^ |
|  | intracellular organelle | Up | 3.82x10^-07^ |
|  | organelle | Up | 3.92x10^-07^ |
|  | eukaryotic translation elongation factor 1 complex | Up | 4.66x10^-06^ |
|  | intracellular | Up | 0.0002 |
|  | large ribosomal subunit | Up | 0.0005 |
|  | myosin filament | Up | 0.0008 |
|  | proton-transporting ATP synthase complex, catalytic core F(1) | Up | 0.0009 |
|  | nucleus | Down | 2.45x10^-30^ |
|  | membrane-bounded organelle | Down | 4.74x10^-18^ |
|  | intracellular membrane-bounded organelle | Down | 4.74x10^-18^ |
|  | intracellular | Down | 7.22x10^-12^ |
|  | intracellular organelle | Down | 8.23x10^-12^ |
|  | organelle | Down | 9.53x10^-12^ |
|  | intracellular part | Down | 4.24x10^-09^ |
